# Resistance to Anti-Angiogenic Therapy in Cancer—Alterations to Anti-VEGF Pathway

**DOI:** 10.3390/ijms19041232

**Published:** 2018-04-18

**Authors:** Yoshiro Itatani, Kenji Kawada, Takamasa Yamamoto, Yoshiharu Sakai

**Affiliations:** 1 Department of Surgery, Graduate School of Medicine, Kyoto University, Kyoto 606-8507, Japan; itatani@kuhp.kyoto-u.ac.jp (Y.I.); masayama@kuhp.kyoto-u.ac.jp (T.Y.); ysakai@kuhp.kyoto-u.ac.jp (Y.S.); 2 Moores Cancer Center, University of California San Diego, San Diego, CA 92093, USA

**Keywords:** anti-angiogenic therapy, resistance to anti-VEGF, tumor microenvironment

## Abstract

Anti-angiogenic therapy is one of the promising strategies for many types of solid cancers. Bevacizumab (Avastin), a recombinant humanized monoclonal antibody of vascular endothelial growth factor (VEGF) A, was approved for the first time as an anti-angiogenic drug for the treatment of metastatic colorectal cancer (CRC) by the Food and Drug Administration (FDA) in 2004. In addition, the other VEGF pathway inhibitors including small molecule tyrosine kinase inhibitors (sunitinib, sorafenib, and pazopanib), a soluble VEGF decoy receptor (aflibercept), and a humanized monoclonal antibody of VEGF receptor 2 (VEGFR2) (ramucirumab) have been approved for cancer therapy. Although many types of VEGF pathway inhibitors can improve survival in most cancer patients, some patients have little or no beneficial effect from them. The primary or acquired resistance towards many oncological drugs, including anti-VEGF inhibitors, is a common problem in cancer treatment. This review summarizes the proposed alternative mechanisms of angiogenesis other than the VEGF pathway. These mechanisms are involved in the development of resistance to anti-VEGF therapies in cancer patients.

## 1. Introduction

**Figure 1:**
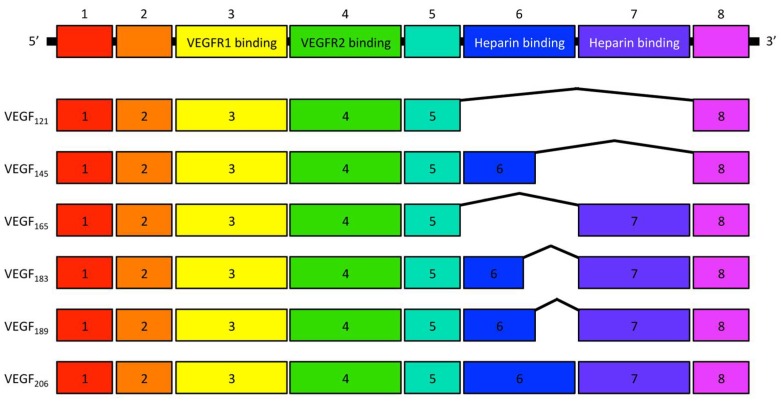
Schematic representation of the VEGFA isoforms. Each number indicates the exon composition and the isoforms consist of splicing variants of these exons from the *VEGFA* gene.

In 1971, Judah Folkman for the first time highlighted that angiogenesis is an essential process for the growth and proliferation of solid tumors [1], which resulted in a notion that anti-angiogenesis might be a potential therapeutic approach against various cancers [1,2,3]. Thereafter, several molecules were identified as angiogenic factors, such as acidic and basic fibroblast growth factors (aFGF and bFGF), angiogenin, and transforming growth factor-α and -β (TGF-α and TGF-β). In 1989, vascular endothelial growth factor (VEGF) A was isolated and cloned [4,5], which led to great progress in understanding the angiogenic mechanisms. VEGFA is a growth/survival factor for endothelial cells and binds to two receptor tyrosine kinases (RTKs), VEGF receptor (VEGFR) 1 and 2 [6]. VEGFA has six isoforms—namely, VEGF_121_, VEGF_145_, VEGF_165_, VEGF_183_, VEGF_189_, and VEGF_206_—that are the resultant variants of alternative splicing of a single, 8-exon *VEGFA* gene [6] (Figure 1). Among these, VEGF_121_ and VEGF_165_ are the two major isoforms. VEGF_121_ binds solely to VEGFR1 and VEGFR2, whereas VEGF_165_ binds to the co-receptors neuropilin (NRP)-1 and -2 via its basic sequence encoded in exon 7, which enhances the binding of VEGF_165_ to VEGFR2 and promotes its bioactivity [7]. As for the receptors, VEGFR2 is expressed on endothelial cells whereas VEGFR1 is expressed on endothelial cells and other cell types, such as smooth muscle cells, fibroblasts, myeloid progenitors, macrophages, and various types of cancer cells [8]. Although the angiogenic effect of VEGFA is predominantly mediated by VEGFR2, VEGFR1 signaling plays a role in tumor cell survival and growth [9,10,11].

In 1993, a monoclonal neutralizing antibody against VEGFA was reported to inhibit tumor growth in the in vivo xenograft model [12]. This idea led to the development of bevacizumab (Avastin), a recombinant humanized monoclonal antibody specific to VEGFA. In 2004, bevacizumab was approved by the U.S. Food and Drug Administration (FDA) for the treatment of metastatic colorectal cancer (CRC) [13]. In addition, various other inhibitors of the VEGF signaling pathway have been developed. The RTK inhibitors (RTKIs) sunitinib (Sutent) [14], sorafenib (Nexavar) [15], and pazopanib (Votrient) [16] are currently approved for the treatment of various types of cancers. Aflibercept (Zaltrap), a soluble recombinant fusion protein that consists of the extracellular domains of VEGFR1 and VEGR2 fused to the Fc portion of human IgG1, neutralizes VEGFA, VEGFB, and placental growth factor (PlGF), and was approved in 2012 by the FDA for the treatment of metastatic CRC [17]. Ramucirumab (Cyramza) is also a monoclonal antibody that binds VEGFR2 to block the VEGF signaling pathway and has been approved by the FDA for the treatment of several types of solid cancers [18].

Despite a large amount of promising data from animal experiments, simply blocking the VEGF signaling pathway by an anti-VEGF monotherapy appears to be ineffective for advanced cases in the clinical setting [19]. This primary or de novo treatment resistance is a common problem in the treatment of cancer patients, even with the most recent sophisticated drugs.

Resistance to anti-VEGF therapy often occurs owing to the escape mechanisms of the angiogenic process through the activation of signaling pathways other than the VEGF pathway. Moreover, it has been proposed that the inhibition of VEGFR by RTKI or an antibody promotes tumor invasiveness and metastasis [20,21]. In this review, we summarize the proposed alternative pathways that are involved in the emergence of resistance to anti-VEGF therapy in cancer.

## 2. Alternative Angiogenic Pathways to the VEGF Pathway That Influence Anti-VEGF Treatment

**Figure 2:**
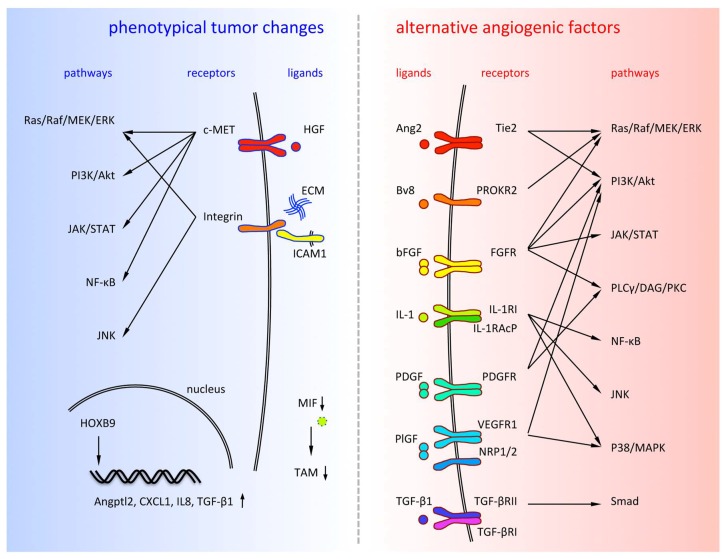
Alternative angiogenic factors are listed on the right side and phenotypical tumor changes are listed on the left side.

Although the VEGF pathway induces the most profound angiogenesis during tumor formation, the prediction of the existence of alternative angiogenic pathways is relevant as we observe various anti-VEGF resistant cancers. In this section, we discuss the potential angiogenic factors that are proposed to contribute to the escape from anti-VEGF treatment (Figure 2, right).

### 2.1. Angiopoietin-2 (Ang2)

Angiopoietin–Tie signaling is a vascular-specific RTK pathway that is essential for blood vessel development, remodeling, and regulation of vascular permeability. Angiopoietin-1 (Ang1) was initially identified as an agonist of the Tie2 receptor, activating this pathway; angiopoietin-2 (Ang2) was identified as an antagonist of the Tie2 receptor [22]. Ang1 affords maturation or stabilization of blood vessels through Tie2, which can be blocked by Ang2, while such inhibition by Ang2 results in the remodeling or initiation of vascular sprouts in the context of VEGF exposure [23]. The Tie2 receptor is expressed on endothelial cells of the blood and lymphatic vessels, the M2 subpopulation of monocytes/macrophages, and hematopoietic stem cells. The Tie2 receptor regulates downstream signaling pathways such as phosphoinositide 3-kinase (PI3K)/Akt and/or mitogen-activated protein kinase (MAPK)/extracellular-related kinase (ERK) (also known as Ras/Raf/MEK/ERK) [24,25]. The Ang/Tie system plays a crucial role in the pathophysiology of the tumor vasculature, as well as normal vasculature, and Ang2 expression is found to be upregulated in many types of cancers [26,27,28,29,30]. Moreover, CRC patients with high serum Ang2 levels exhibited poor response to bevacizumab treatment, suggesting that Ang2 plays an important role in the resistance mechanism against anti-VEGF therapy [31]. In preclinical settings, dual blockade of VEGF and Ang2 suppressed the revascularization and tumor progression of anti-VEGF therapy-resistant cancers [32,33,34,35]. Some clinical trials are underway and their results regarding the efficacy of vanucizumab, a humanized bi-specific monoclonal antibody against two different targets—VEGF-A and Ang2—are now pending [36,37].

### 2.2. Bombina Variegata Peptide 8 (Bv8)

Bv8, also known as prokineticin-2, was initially purified from the skin secretion of the yellow-bellied toad, *Bombina variegata*. Bv8 and endocrine gland-derived VEGF (EG-VEGF, also known as prokineticin-1) belong to the same family of proteins and bind to prokineticin receptor (PROKR)-1 and -2 (G-protein coupled receptors) to activate the downstream MAPK/ERK pathway [38,39]. They induce angiogenesis by stimulating proliferation, migration, and survival of vascular endothelial cells [40]. Although Bv8 is mainly expressed in the testis, it is also expressed in bone marrow. Bv8 is highly expressed in the neutrophil population under the control of granulocyte colony-stimulating factor (G-CSF) stimulated by signal transducer and activator of transcription 3 (STAT3) signaling in the bone marrow or inside the tumor microenvironment [41,42]. It is well established that inflammation strongly promotes the initiation of several types of cancers, such as CRC following inflammatory bowel disease, liver cancer following hepatitis, gastric cancer following gastritis, esophageal cancer following esophagitis, and so forth. It was reported that the inflammatory cytokine interleukin-17 (IL-17)-producing T helper type 17 (T_H_17) cells initiate a paracrine network to confer resistance to anti-VEGF therapy [43]. G-CSF and its upstream cytokine IL-17A are the main players of inflammation; thus, it is conceivable that G-CSF is abundant in tumor tissues [44]. Indeed, serum G-CSF levels in CRC patients are higher than those in healthy volunteers and are associated with the stage of tumor progression [45,46].

It is known that anti-VEGF therapy induces tumor-associated neutrophil (TAN) infiltration into the tumor microenvironment, which would be a predictive biomarker for patients treated with bevacizumab [47,48,49,50,51,52]. These observations led to the discovery that neutrophils provide the resistance mechanisms against anti-VEGF therapy [53]. In brief, tumor cells or tumor tissues secrete G-CSF during the inflammatory conditions and/or in the cytokine-rich environment through nuclear factor-κB (NF-κB) signaling driven by IL-17A [43]. It is also known that the oncogenic Ras pathway promotes G-CSF expression through the activation of its downstream MEK/ERK signaling [54]. Next, G-CSF promotes the recruitment of neutrophils into the tumor site, stimulates them to express Bv8, and promotes angiogenesis, which results in the escape from anti-VEGF therapy [55,56,57]. PKRA7, a small molecule Bv8 antagonist, could suppress tumor formation in vivo by inhibiting angiogenesis and infiltration of myeloid-derived cells [58]. Neutralization of Bv8 and its upstream G-CSF by using monoclonal antibodies was also effective in tumor suppression [41]. To date, effective regimens using Bv8 inhibitors in combination with or without other anti-angiogenic reagents are pending in clinical trials.

### 2.3. Fibroblast Growth Factor (FGF)

The FGF family consists of 22 members. Among them, 18 are secretory proteins that bind to four types of RTK–FGF receptor (FGFR), i.e., FGFR1–4, whereas four of them are intracellular non-signaling proteins that serve as cofactors of voltage-gated sodium channels [59]. FGF/FGFR signaling is essential in the earliest stage of embryonic development and in the maintenance of adult tissues by stimulating cell proliferation, survival, migration, differentiation, and metabolism of the target cells. Binding of FGF to FGFR tyrosine kinase activates the downstream pathways such as MAPK/ERK, PI3K/Akt, STAT, and/or diacylglycerol (DAG)/protein kinase C (PKC) and the inositol triphosphate (IP3)-Ca^2+^ signaling cascade via phospholipase-Cγ (PLCγ) activation [60,61,62,63]. In addition to normal tissue development and homeostasis, FGF/FGFR signaling plays crucial roles in cancer development and progression [64,65]. FGFR is expressed on cancer cells as well as several types of stromal cells, such as cancer-associated fibroblasts (CAFs), endothelial cells, and tumor-infiltrating myeloid cells [65]. Angiogenesis is one of the key mechanisms of FGF/FGFR signaling during tumor progression, and the upregulation of FGF2 (also known as bFGF) is observed in anti-VEGF-resistant tumors, especially in tumors that are exposed to a hypoxic environment [66,67,68,69]. Several preclinical studies demonstrated the benefit of dual blockade of VEGF and FGF signaling pathways during cancer treatment [66,70,71,72,73]. These data encouraged us to further study the FGF/FGFR inhibitors against anti-VEGF-resistant tumors. Unfortunately, so far, small molecule tyrosine kinase inhibitors of VEGF, as well as FGF receptors (dovitinib used to inhibit VEGFR and FGFR; and nintedanib used to inhibit VEGFR, FGFR, and platelet-derived growth factor receptor (PDGFR)) were not effective in treating cancer patients after recurrence following anti-VEGF therapy [74,75].

### 2.4. Interleukin-1 (IL-1)

IL-1 is a family of 11 cytokines that affect tumor progression, as well as inflammatory processes. It includes receptor agonists (IL-1α, IL-1β, IL-18, IL-33, IL-36a, IL-36b, IL-36γ, and IL-37) and antagonists (IL-1Ra, IL-36Ra, and IL-38). Among these 11 cytokines, IL-1α and IL-1β induce the ability of tumor cells to initiate and complete the angiogenic process [76]. IL-1α and IL-1β bind to the same receptor, i.e., type 1 IL-1 receptor (IL-1R1), to activate the downstream signaling, whereas IL-1Ra inhibits IL-1R1 in a competitive binding manner. They initially bind to the extracellular chain of IL-1R1 to recruit its co-receptor IL-1 receptor accessory protein (IL-1RAcP) that is needed to activate the signal transduction of NF-κB, c-Jun N-terminal kinase (JNK), and p38 MAPK [77]. IL-1β was reported to induce the in vivo production of angiogenic factors, such as hypoxia-inducible factor 1α (HIF-1α), VEGF, and C-X-C motif chemokine (CXC) ligand 2 (CXCL2), which results in the rapid growth of tumor cells accompanied by hyperneovascularization [78,79]. IL-1α and IL-1β were upregulated in a mouse model with pancreatic cancer resistant to anti-VEGF therapy, suggesting that they play an important role in the resistance mechanism to anti-VEGF therapy [80]. In vivo experiments demonstrated that the neutralization of IL-1, as well as other candidate molecules such as CXC receptor (CXCR) 1/2 and TGF-β signaling, abrogated resistance to anti-VEGF therapy in a murine model of pancreatic cancer [81]. Clinical trials for dual blockade of IL-1 and VEGF signaling pathways are still pending.

### 2.5. Platelet-Derived Growth Factor (PDGF)

During embryonic development and tissue repair, the PDGF family members play important roles in cell growth, survival, and motility of mesenchymal cells and other cell types [82]. The PDGF family consists of homodimers of PDGF-AA, -BB, -CC, -DD, and the heterodimer of PDGF-AB. They bind to tyrosine kinase PDGFR, which consists of α and β isoforms, and they dimerize upon binding to the ligand dimers to activate downstream signal transduction, such as PI3K and PLCγ [83]. It is well known that a constitutively active PDGFR mutation contributes to the formation of a gastrointestinal stromal tumor (GIST). Moreover, the overactivation of PDGF signaling in the tumor microenvironment promotes tumor growth through angiogenesis [84]. Among the PDGF family members, PDGF-C was upregulated in CAFs infiltrating into anti-VEGF-resistant tumors in vivo [85]. In this model, a PDGF-C neutralizing antibody suppressed CAF-mediated tumor progression, indicating an additional effect upon anti-VEGF antibody [85].

Sunitinib is a multi-tyrosine kinase inhibitor that blocks VEGFR and PDGFR, as well as c-Kit, fms-like tyrosine kinase 3 (FLT-3), and colony stimulating factor-1 receptor (CSF-1R) [86]. In 2006, sunitinib was first approved by the FDA for the treatment of imatinib-resistant GIST (second-line setting) and metastatic renal cell carcinoma (RCC) (first-line setting); and later, in 2017, its application was expanded to the adjuvant treatment of RCC [87,88]. Despite the success of the dual blockade of VEGFR and PDGFR by sunitinib, a combination strategy using bevacizumab (to block VEGF signaling) and imatinib (to block PDGF signaling) was not effective but was toxic during RCC treatment [89]. Further investigation is required to clearly understand the necessity of dual blockade of these pathways in clinical settings.

### 2.6. Placental Growth Factor (PlGF)

PlGF is a member of the VEGF subfamily and binds to VEGFR1 and its co-receptors NRP-1 and -2, but not VEGFR2 [90,91]. PlGF stimulates growth, survival, and migration of endothelial cells, macrophages, and bone marrow progenitors, as well as tumor cells via VEGFR1 and its downstream PI3K/Akt and p38 MAPK pathways independent of VEGFA signaling [92,93]. Several observations have reported the upregulation of PlGF in patients treated with anti-VEGF therapy, suggesting that PlGF might be a therapeutic target for anti-angiogenic treatment-resistant tumors [68,94,95,96]. It was reported that PlGF knockout (*Pgf*^−/−^) mice exhibited normal embryonic angiogenesis and that *Pgf*^−/−^ mice subjected to ischemia, wound healing, or tumor burden conditions exhibited impaired pathological angiogenesis [97]. These findings led to the idea that PlGF blockade might inhibit pathological angiogenesis without affecting healthy blood vessels [98]. Unfortunately, so far, anti-PlGF neutralizing antibodies in combination with anti-VEGF antibodies appear to have minimal effect on tumor suppression in vivo [99]. Moreover, its phase I clinical study in combination with bevacizumab demonstrated no improvement in recurrent glioblastoma patients compared to single-agent bevacizumab treatment, although the anti-PlGF antibody toxicity was acceptable and manageable [100].

In 2012, aflibercept (also known as ziv-aflibercept in the U.S.), a soluble VEGF decoy receptor, was approved by the FDA for the treatment of metastatic CRC in combination with 5-fluorouracil, leucovorin, and irinotecan [101]. It is a recombinant protein that consists of the extracellular domain of VEGFR1 and 2 and the Fc portion of human IgG1. Owing to its structure, ziv-aflibercept neutralizes both VEGF and PlGF [102]. In patient-derived xenograft (PDX) models, ziv-aflibercept exhibited higher tumor suppressive activity than bevacizumab [103]. Further evaluation regarding the anti-PlGF activity in addition to anti-VEGF is needed in clinical settings.

### 2.7. Transforming Growth Factor-β (TGF-β) Signaling

TGF-β signaling is a highly conserved pathway that regulates several cellular processes including growth, differentiation, and apoptosis [104]. The TGF-β superfamily consists of two major branches: TGF-β/Activin and bone morphogenetic protein (BMP). Upon ligand binding, type II receptors activate type I receptors to initiate Smad transcription factors by phosphorylating receptor-regulated Smads (R-Smads, Smad2/3 in the TGF-β/Activin pathway, and Smad1/5 in the BMP pathway), which form a complex with the common partner Smad (Co-Smad, Smad4), and work as transcription factors [105]. TGF-β1 induces angiogenesis either directly or by activating fibroblasts to produce extracellular matrix (ECM) adhesion and stimulating the tube formation of endothelial cells [106,107,108]. Although TGF-β signaling causes tumor suppressive effects during the early stage, it switches toward malignant conversion and tumor progression at later stages [109,110]. Many types of tumor tissues express higher levels of TGF-β compared to the adjacent normal tissues, and its expression levels are correlated with patient survival [111,112,113]. Anti-VEGF therapy-resistant tumors sometimes exhibit high levels of TGF-β1 expression, suggesting that it might play an important role in the acquired resistance to anti-angiogenic therapy [114]. Despite the abundant evidence showing the angiogenic function of TGF-β signaling and the synergistic effect of TGF-β and VEGF signaling in cancer progression, there seem to be few clinical trials showing the combined effect of blocking both TGF-β and VEGF signaling pathways.

## 3. Phenotypical Changes of Tumor Cells during Anti-Angiogenic Therapy

Anti-angiogenic therapy induces vascular regression, which leads to intratumoral hypoxia and selection of more invasive cancer cells that are resistant to anti-angiogenic therapy. It is well known that hypoxic conditions induce upregulation of VEGF expression at a transcriptional level through its upstream transcription factor HIF-1 [115]. This finding indicates that the anti-angiogenic therapy is ineffective without blocking VEGF signaling. Moreover, HIF-1 was proposed to have several functions that promote cancer cell survival in the hypoxic environment. In this section, we discuss the proposed tumor factors that are altered during the development of resistance to anti-angiogenic therapy in cancers (Figure 2, left).

### 3.1. Hepatocyte Growth Factor (HGF)/Tyrosine Protein Kinase Met (c-MET) Pathway

c-MET signaling in the presence of its ligand HGF controls tumor growth and invasiveness by activating MAPK/ERK cascades, PI3K/Akt axis, STAT3 pathway, and/or NF-κB inhibitor-α kinase (IKK)—NF-κB complex [116,117,118]. It is one of the most investigated signaling pathways in anti-VEGF therapy-resistant tumors. In glioblastoma patients, c-MET expression was highly upregulated during the recurrence after bevacizumab treatment, which was not observed in patients without bevacizumab treatment [119]. It was reported that *c-MET* transcription is promoted under hypoxic conditions via the direct regulation of HIF-1 [116]. Moreover, VEGF was reported to negatively regulate c-MET activation, resulting in the direct suppression of tumor invasion in a mouse model of glioblastoma [120]. These findings suggest a hypothesis that the neutralization of VEGF by bevacizumab might promote c-MET protein expression and also remove suppression of c-MET phosphorylation through VEGF, resulting in the dual activation of c-MET signaling. Unfortunately, the c-MET inhibitor onartuzumab did not exhibit any clinical benefit when administered in combination with a bevacizumab regimen to advanced non-small cell lung cancer (NSCLC) patients [121].

### 3.2. Homeobox B9 (HOXB9)

*HOX* genes encode highly conserved transcription factors and play crucial roles in embryonic development and oncogenesis, as well as tumor suppression [122]. HOXB9 is one of the HOX superfamily members and is upregulated in many types of cancers [123,124,125,126]. It controls the expression of some angiogenic factors, such as angiopoietin-like 2 (Angptl2), CXCL1, IL8, and TGF-β1, which causes resistance to bevacizumab treatment in mouse xenograft models [123,127]. Importantly, HOXB9 protein expression might be a predictive biomarker for metastatic CRC patients treated with bevacizumab [127]. Although the mechanism of how some of the tumors exhibit high HOXB9 expression remains unidentified, HOXB9 silencing in the bevacizumab-resistant xenograft model significantly decreased the expression of alternative angiogenic factors, causing the model to become sensitive to bevacizumab, and resulting in prolonged survival in vivo [127]. Further clinical studies are needed to validate whether HOXB9 can be a potential therapeutic target for anti-VEGF therapy-resistant tumors.

### 3.3. Integrin

Integrins are transmembrane receptors that play important roles in cell–cell and cell–ECM adhesion. They are heterodimers formed by the combination of α and β subunits. Upon binding to ECM as their ligand, they induce signal transduction pathways that mediate cytoskeletal organization, cell cycle regulation, cell survival, and proliferation under both normal and pathological states via MAPK/ERK and JNK pathways [128]. Normal host cells in the tumor microenvironment express integrins, which promote angiogenesis and lymphangiogenesis [129,130]. In addition to the host cells in the tumor microenvironment, tumor cells also exhibit high expression of integrins during malignant progression, and their expression levels are correlated with disease progression and poor survival of patients [131,132,133]. Among the members, β1 integrin is implicated in resistance to cancer treatment [134,135,136]. Indeed, in some contexts, β1 integrin is upregulated in the clinical specimens of bevacizumab-resistant glioblastomas [137]. HIF-1α, induced by the hypoxic microenvironment, generated during anti-angiogenic therapy stimulates β1 integrin expression, which interacts with c-MET signaling and results in an enhancement of tumor cell invasiveness [138,139,140]. Preclinical studies of glioblastoma xenograft models in vivo demonstrated the advantage of β1 integrin inhibition in bevacizumab-resistant tumors, as well as non-resistant tumors [138,141].

### 3.4. Intracellular Cell Adhesion Molecule 1 (ICAM-1)

ICAM-1, also known as CD54, was reported to be overexpressed in bevacizumab-resistant glioblastomas in a mouse xenograft model [142]. ICAM-1 plays a key role as an adhesion molecule by binding to two types of integrins: lymphocyte function-associated antigen-1 (LFA-1, also known as CD11a/CD18) and macrophage antigen-1 (Mac-1, also known as CD11b/CD18) [143]. When the glioma stem cell line GSC11 was subjected to hypoxic conditions, HIF-1-induced phosphorylated STAT3 activated ICAM-1 transcription and promoted macrophage infiltration into the tumor tissues [142]. When ICAM-1 expression in cancer cells was knocked down by shRNA, tumor growth and invasion were significantly suppressed and mice implanted with these cells exhibited improved survival [142].

### 3.5. Macrophage Migration Inhibitory Factor (MIF)

MIF is classified as an inflammatory cytokine that regulates macrophage function through the suppression of their anti-inflammatory activity. Tumor-associated macrophages (TAMs), mainly M2-polarized macrophages, promote tumor progression by stimulating angiogenesis and tumor cell migration/invasion, as well as suppressing tumor immunity [144]. In the tissue specimens of bevacizumab-resistant glioblastoma patients, MIF expression was decreased and TAM infiltration was increased compared to those in bevacizumab-sensitive ones [145]. As VEGF increases MIF production in a VEGFR-dependent manner, inhibition of the VEGF pathway directly depletes MIF expression, resulting in TAM recruitment and M2 polarization in bevacizumab-resistant glioblastoma patients. Glioblastoma xenograft tumors transduced with MIF expression grew slowly and exhibited low TAM infiltration in vivo [145]. The application of this target in clinical settings is still pending.

## 4. Discussion

The tumor microenvironment is as important as the tumor cells and is a major component of tumor tissues. It consists of normal host immune cells, bone marrow-derived inflammatory cells, blood vessels, lymphatic vessels, fibroblasts, and ECM. Neutralization of VEGF by using bevacizumab is a pioneering approach to targeting the tumor microenvironment during cancer therapy. The recent success of cancer immunotherapy using immune checkpoint inhibitors is also designed to target the tumor microenvironment. For example, nivolumab, pembrolizumab, and pidilizumab are against programmed cell death protein 1 (PD-1), atezolizumab and avelumab are against programmed death-ligand 1 (PD-L1), and ipilimumab is against cytotoxic T-lymphocyte-associated protein 4 (CTLA-4). As described above, host immune cells such as TANs and TAMs contribute to some of the proposed mechanisms of anti-VEGF therapy resistance [47,48,52,145]. Infiltration of CD8+ cytotoxic T-lymphocytes (CTLs), also known as “immunoscore,” is a good prognostic biomarker for cancer patients [146,147,148]. Similarly, RCC specimens treated with anti-angiogenic therapy exhibited infiltration of CD4+ and forkhead box P3 (FOXP3)+ regulatory T cells (T-reg). A high T-reg infiltration has a significant correlation with poor overall survival [149]. These findings suggest the importance of combination therapy using anti-angiogenic drugs and immune checkpoint inhibitors. Currently, several clinical trials are ongoing, which might lead to a new era of anti-angiogenic therapy [150].

Even after 10 years of approval by the FDA of the first anti-VEGF drug, i.e., bevacizumab, resistance to anti-VEGF therapy remains a challenge in the treatment of cancer patients. So far, the mechanisms of resistance development are not completely unveiled. In addition to the immune checkpoint inhibitors, other potential therapeutic agents appear to be available that need to be clinically validated as treatment strategies for anti-VEGF therapy-resistant tumors.

## Abbreviations

Ang2	angiopoietin-2
BMP	bone morphogenetic protein
Bv8	*Bombina variegata* peptide 8
CRC	colorectal cancer
CSF-1R	colony-stimulating factor-1 receptor
CTL	cytotoxic T-lymphocyte
CTLA-4	cytotoxic T-lymphocyte-associated protein 4
CXC	C-X-C motif chemokine
CXCL	CXC ligand
CXCR	CXC receptor
DAG	diacylglycerol
ECM	extracellular matrix
EGFL	epidermal growth factor-like domain
EG-VEGF	endocrine gland-derived vascular endothelial growth factor
ERK	extracellular-related kinase
FDA	Food and Drug Administration
FGF	fibroblast growth factor
FGFR	FGF receptor
FLT-3	fms-like tyrosine kinase 3
FOXP3	forkhead box P3
G-CSF	granulocyte colony-stimulating factor
GIST	gastrointestinal stromal tumor
HIF-1	hypoxia-inducible factor 1
HOX	homeobox
IKK	nuclear factor-κB inhibitor-α kinase
IL	interleukin
IL-1R1	type 1 IL-1 receptor
IL-1RAcP	IL-1 receptor accessory protein
IP3	inositol triphosphate
LFA-1	lymphocyte function-associated antigen-1
JNK	c-Jun N-terminal kinase
MAPK	mitogen-activated protein kinase
Mac-1	macrophage antigen-1
MIF	macrophage migration inhibitory factor
NF-κB	nuclear factor-κB
NRP	neuropilin
NSCLC	non-small cell lung cancer
PD-1	programmed cell death protein 1
PD-L1	programmed death-ligand 1
PDGF	platelet-derived growth factor
PDGFR	platelet-derived growth factor receptor
PI3K	phosphoinositide 3-kinase
PKC	protein kinase C
PLC γ	phospholipase C γ
PlGF	placental growth factor
PROKR	prokineticin receptor
RCC	renal cell carcinoma
RTK	receptor tyrosine kinase
RTKI	RTK inhibitor
STAT	signal transducer and activator of transcription
TAN	tumor-associated neutrophil
T-reg	regulatory T cells
TAM	tumor-associated macrophage
TGF	transforming growth factor
T_H_17	T helper type 17
VEGF	vascular endothelial growth factor
VEGFR	VEGF receptor
